# Effect of Oral Hypoglycaemic Agents on Carotid Artery Intima‐Media Thickness in Patients With Cardiovascular Disease and/or Diabetes—A Systematic Review

**DOI:** 10.1002/edm2.70140

**Published:** 2025-11-20

**Authors:** Ali Shabu, Syed Mohammad Naqvi, Farshad Hesari, Syed Yaseen Naqvi, Wael Tawfick

**Affiliations:** ^1^ National Institute for Prevention and Cardiovascular Health (NIPC) University of Galway Galway Ireland; ^2^ Faculty of Education and Health Sciences University of Limerick Limerick Ireland; ^3^ Centre for Languages and Literature Lund University Lund Sweden

**Keywords:** cardiovascular disease, carotid artery intima‐media thickness, diabetes mellitus, DPP‐4 inhibitors, metformin, oral hypoglycaemic agents, SGLT‐2 inhibitors, sulfonylureas, thiazolidinediones

## Abstract

**Introduction:**

Atherosclerotic cardiovascular disease (ASCVD) is a global concern, with diabetes being a key risk factor. Preventive measures increasingly rely on surrogate markers, such as carotid artery intima‐media thickness (CIMT), a marker linked to ASCVD. Given the connection between dysglycaemia and ASCVD, the impact of oral hypoglycaemic agents (OHA) on CIMT is of interest. Despite the cardiovascular benefits of several OHAs, their effect on CIMT remains uncertain. This review aims to clarify the influence of OHAs on CIMT in patients with ASCVD and/or diabetes.

**Objectives:**

This systematic review aims to assess the effect of OHAs on CIMT in patients with ASCVD and/or diabetes mellitus (Type 1 or Type 2). We aim to provide evidence on the role of OHAs in reducing cardiovascular events in these high‐risk patients.

**Methodology:**

A systematic search of databases, including Cochrane, Embase, CINAHL, Scopus and PubMed, was conducted to identify relevant randomised controlled trials (RCTs). We analysed the efficacy and adverse effects of OHAs on CIMT in adults with diabetes and/or cardiovascular disease.

**Results:**

The initial search identified 629 studies, with 13 selected, involving 3849 participants. Pioglitazone showed effectiveness in slowing CIMT progression in two out of three studies. Repaglinide was effective in reducing CIMT and inflammation, while Rosiglitazone showed no significant effect. Metformin, Sitagliptin and Alogliptin studies yielded mixed results, with some showing reduced CIMT progression but increased gastrointestinal and hypoglycaemia risks. SGLT‐2 inhibitors Tofogliflozin and Ipragliflozin showed no significant CIMT reduction.

**Conclusion:**

The study suggests that prolonged use of Pioglitazone, Repaglinide and Alogliptin may significantly slow CIMT progression, improving cardiovascular risk management in patients with diabetes and/or cardiovascular disease. Further research is needed to understand the benefits and optimise oral hypoglycaemic treatment strategies for these patients.

## Graphical abstract

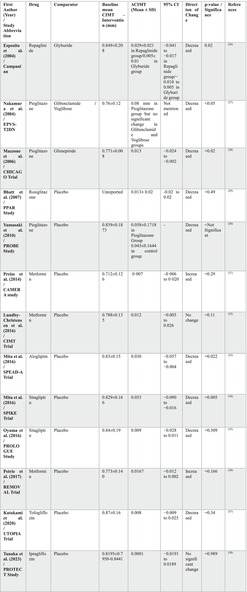

## 1 Introduction

### 1.1 Description of the Condition

Atherosclerotic cardiovascular disease (ASCVD) is a chronic inflammatory condition of the arteries and a leading cause of morbidity and mortality, accounting for around 85% of deaths in the Western world [1, 2]. ASCVD often remains asymptomatic until serious events like myocardial infarction (MI) or stroke occur. Its clinical manifestations vary based on the affected vascular bed, including MI, ischaemic heart disease, cerebrovascular events and peripheral arterial disease [3]. Well‐established risk factors for ASCVD include smoking, hypertension, diabetes, dyslipidaemia, genetic predisposition, age and male gender [4].

The pathophysiology of ASCVD is complex, involving lipid accumulation, immune cell activity and fibrous tissue deposition in the arterial walls [5]. Endothelial dysfunction, triggered by factors such as hyperlipidaemia and oxidative stress, initiates atherosclerosis [6]. This leads to the formation of fatty streaks through the infiltration of monocytes, their transformation into foam cells and the progression to fibrous plaques [7, 8]. Chronic inflammation and smooth muscle proliferation exacerbate the disease [9].

While advanced ASCVD is clinically apparent, it begins insidiously, often with subclinical atherosclerosis. Carotid artery intima‐media thickness (CIMT) is a non‐invasive ultrasound measurement of the carotid artery wall [10]. An increased CIMT reflects early pathological changes, including endothelial dysfunction, smooth muscle cell migration and inflammation, which are precursors to plaque formation [10, 11, 12, 13]. This makes it a valuable tool for subclinical disease detection and cardiovascular risk stratification.

### 1.2 Standard Care and Description of the Intervention

The management of ASCVD and DM primarily involves lifestyle modification and pharmacological interventions. Among the pharmacological options, hypoglycaemic agents play a crucial role in controlling blood glucose levels, thereby reducing the risk of complications associated with hyperglycaemia. Hyperglycaemia is a well‐established risk factor for atherosclerosis, making the management of blood glucose particularly important in patients with co‐existing ASCVD and diabetes [14].

Established oral hypoglycaemic agents (OHA) have been available for many years and yielded positive results; however, with the advent of novel OHAs, such as dipeptidyl peptidase‐4 inhibitors (DPP‐4i), sodium‐glucose cotransporter‐2 inhibitors (SGLT‐2i) and peroxisome proliferator‐activated receptor gamma (PPARγ) agonists, has further developed diabetes treatment guidelines [15]. These newer agents are recognised not only for their efficacy in glycaemic control but also for their significant cardiovascular benefits. In particular, SGLT‐2is have gained prominence in clinical guidelines for patients with diabetes who are at high risk for cardiovascular events, reflecting their multifaceted effects beyond glucose regulation [16, 17].

The use of these OHAs in patients with diabetes and ASCVD is essential for managing both conditions effectively. The European Society of Cardiology (ESC) specifically recommends SGLT‐2is for this patient population due to their ability to reduce the risk of major adverse cardiovascular events (MACE), such as myocardial infarction and stroke [18]. Despite these advancements, the impact of OHAs on CIMT remains underexplored. CIMT measurement is a non‐invasive method used to assess subclinical atherosclerosis, making it a valuable tool for evaluating cardiovascular risk in clinical and research settings [19]. While traditional and novel OHAs have shown cardiovascular benefits, their specific effects on CIMT in patients with ASCVD and/or DM have not been thoroughly investigated.

OHAs operate through various mechanisms, such as enhancing insulin sensitivity (e.g., metformin), increasing insulin secretion (e.g., sulfonylureas) or reducing glucose reabsorption in the kidneys (e.g., SGLT‐2is) [20]. As mentioned, some OHAs, such as SGLT‐2is, offer cardiovascular benefits that extend beyond glycaemic control, potentially impacting CIMT and the progression of atherosclerosis.

Given the critical role of CIMT as an early indicator of atherosclerosis, a systematic review is warranted to evaluate the effectiveness of different OHAs in reducing CIMT among patients with ASCVD and/or DM. This review will synthesise existing evidence, providing valuable insights for clinicians in selecting the most appropriate OHA for managing cardiovascular risk in this high‐risk patient population. These findings will contribute to improved cardiovascular outcomes, better patient care and potentially lower healthcare costs by refining the use of OHAs in clinical practice.

### 1.3 Importance of the Review

Despite significant advances in understanding the effects of OHAs on cardiovascular outcomes, the impact of different OHAs on CIMT remains unclear. Although some clinical trials have explored the efficacy of specific OHAs in this context, there is a need for a systematic review to assess the effects of various OHAs on CIMT in patients with ASCVD and/or DM [21]. Such a review is essential, as CIMT serves as an early indicator of atherosclerosis and is a predictor of cardiovascular events, making it a crucial outcome for evaluating cardiovascular risk management strategies.

Previous systematic reviews have primarily focused on the broader cardiovascular benefits of hypoglycaemic agents, such as the role of SGLT‐2is in improving survival and mortality in patients with heart failure or enhancing glycaemic control [22]. However, few have specifically examined the long‐term impact of OHAs on CIMT, especially in patients with co‐existing ASCVD and diabetes, a population at particularly elevated risk for adverse cardiovascular events. By concentrating on this specific patient population and outcome, our systematic review aims to fill a significant gap in the current literature.

This review will enable a comprehensive comparison of the relative effectiveness of different OHAs in reducing CIMT, offering valuable insights for clinical decision‐making and the development of more targeted treatment guidelines. Our findings will contribute to improved patient outcomes, including reduced cardiovascular morbidity and mortality, by informing the optimal use of OHAs in managing atherosclerosis risk. Furthermore, this review will identify areas where additional research is needed, paving the way for future studies that can deepen our understanding of the role of OHAs in cardiovascular health. Ultimately, this work will support better management of atherosclerosis and its associated risks, leading to improved patient care and potentially lower healthcare costs.

## 2 Methods

### 2.1 Criteria for Considering Studies for This Review

This review included RCTs with at least one follow‐up measurement of CIMT obtained at 12 months or more beyond the date at which the intervention was initiated.

### 2.2 Types of Studies

RCTs evaluating OHAs efficacy on CIMT progression/reduction in adults diagnosed with ASCVD and/or DM compared to conventional therapy involving other glucose modulating therapies. The studies reported at least one follow‐up ultrasound measurement of CIMT obtained at 12 months or more following the initiation of the intervention as an outcome.

### 2.3 Types of Participants

- The review included adults with ASCVD and/or DM who received treatment with OHAs in isolation or in addition to other cardioprotective therapies and anti‐diabetic medications.
- DM diagnosis was established using values of HbA1c > 6.5%, known documentation of a previous diagnosis, presence of pertinent signs and symptoms or regional diagnostic criteria employed in the jurisdiction.
- ASCVD was defined as the presence of clinical signs or symptoms of clinical atherosclerosis, such as angina, MI, stroke, peripheral arterial disease or any previous cardiovascular or cerebrovascular event, indicating compromise of vascular supply due to atherosclerosis.
- Trials which included treatment‐naive patients with a recent diagnosis of ASCVD and/or T1DM or T2DM receiving their first treatment course were evaluated, with results from these studies reported on.
- Surrogate outcome measures, such as CIMT measurement, carotid plaque score, cholesterol and lipid levels, C‐reactive protein levels, glucose levels, measures of adiposity and adverse event rates were included.
- The review also considered articles that reported on the impact of OHAs on various indices of CIMT progression, and OHA‐associated side effects.
- Furthermore, only RCTs published in the English language were considered for inclusion.

Trials that did not include patients with a confirmed diagnosis of ASCVD and/or T1DM or T2DM, publications without alternative comparator arms/control groups or studies that included the use of non‐oral hypoglycaemic agents as an intervention were excluded.

### 2.4 Types of Interventions

Trials that assessed the efficacy of OHAs in patients with ASCVD and/or DM compared to conventional therapy, which includes insulin, other OHAs, dietary modification, physical activity, weight management, statins and anti‐hypertensives, and inert placebos were considered. A number of OHAs of different classes were evaluated in the RCTs, including Repaglinide (a meglitinide), Glyburide/Glibenclamide (a sulfonylurea), Voglibose (an alpha‐d‐glucosidase inhibitor), Rosiglitazone (a thiazolidinedione), Pioglitazone (a thiazolidinedione), Metformin (a biguanide), Sitagliptin (a DPP‐4 inhibitor), Alogliptin (a DPP‐4 inhibitor), Tofogliflozin (a SGLT‐2 inhibitor) and Ipragliflozin (a SGLT‐2 inhibitor). The intervention may be assessed by means of a randomised double‐blind study comparing the change in mean CIMT between an intervention and a placebo or alternative OHAs or a randomised open‐label trial comparing the change in mean CIMT between an intervention and a placebo or alternative OHAs.

### 2.5 Outcome Measures

CIMT measurements obtained as the average mean value in millimetres (mm) from ultrasound scans of the common carotid artery were used. In RCTs evaluating CIMT at various vessels or segments, the mean average of those values was used. The primary outcome of the study was the CIMT measurements at long‐term follow‐up. The included studies reported CIMT using differing methods, with some presenting absolute values and others reporting change from baseline. To ensure comparability, all values were standardised to millimetres. For studies such as EPVS‐T2DN and CIMT that reported only final CIMT values, mean change was calculated as the difference between baseline and follow‐up measurements. Where change from baseline was directly reported, as in CHICAGO, SPEAD‐A and SPIKE, these data were extracted as published. Standard deviations were derived from confidence intervals where necessary. This standardisation allowed for a consistent interpretation of mean CIMT change across the included randomised controlled trials.

### 2.6 Primary Outcomes

The primary outcome analysed for the purposes of the study was mean change in CIMT at long‐term follow‐up.

### 2.7 Secondary Outcomes

The secondary outcomes of the review focused on long‐term effects of OHA on various health parameters in patients with ASCVD and/or diabetes. These outcomes were categorised into six groups:

1. *Glycaemic control*: Evaluated OHA efficacy on HbA1c, C‐peptide, postprandial glucose, insulin and fasting blood glucose levels.
2. *Metabolic/biochemical parameters*: Assessed changes in lipid profiles (HDL‐c, LDL‐c), body mass index (BMI), waist circumference, blood pressure and pulse rate.
3. *Inflammatory markers*: Monitored systemic vascular inflammation through interleukin‐6 and C‐reactive protein levels.
4. *Renal function*: Analysed eGFR and serum creatinine levels.
5. *Major adverse cardiovascular events (MACE)*: Considered all‐cause mortality from myocardial infarction, stroke, heart failure, cardiovascular death or revascularisation.
6. *Adverse events*: Recorded serious events like hypoglycaemic episodes or other unintended outcomes related to treatment.

### 2.8 Search Methodology

A comprehensive search of five prominent electronic scientific databases (Cochrane Library, Embase, CINAHL, Scopus and PubMed) was performed in accordance with the Preferred Reporting Items for Systematic Reviews and Meta‐Analysis Guidelines [23]. The search strategy was designed to identify relevant articles using the following predefined keywords: ‘Oral Hypoglycaemic Agents, oral anti‐diabetic drugs, metformin, sulfonylureas, DPP‐4 inhibitors, SGLT‐2 inhibitors, thiazolidinediones, Carotid Intima‐Media Thickness, carotid IMT, CIMT’.

### 2.9 Reviewing Supplementary Resources

The lists of cited works were evaluated to identify relevant studies, and article abstracts were examined for possible supplementary links. The review also included retraction notices and related errors observed in the included studies. Additionally, those with expertise in this domain were consulted to gather information on unpublished research and studies currently in progress.

### 2.10 Data Acquisition and Evaluation

**FIGURE 1:**
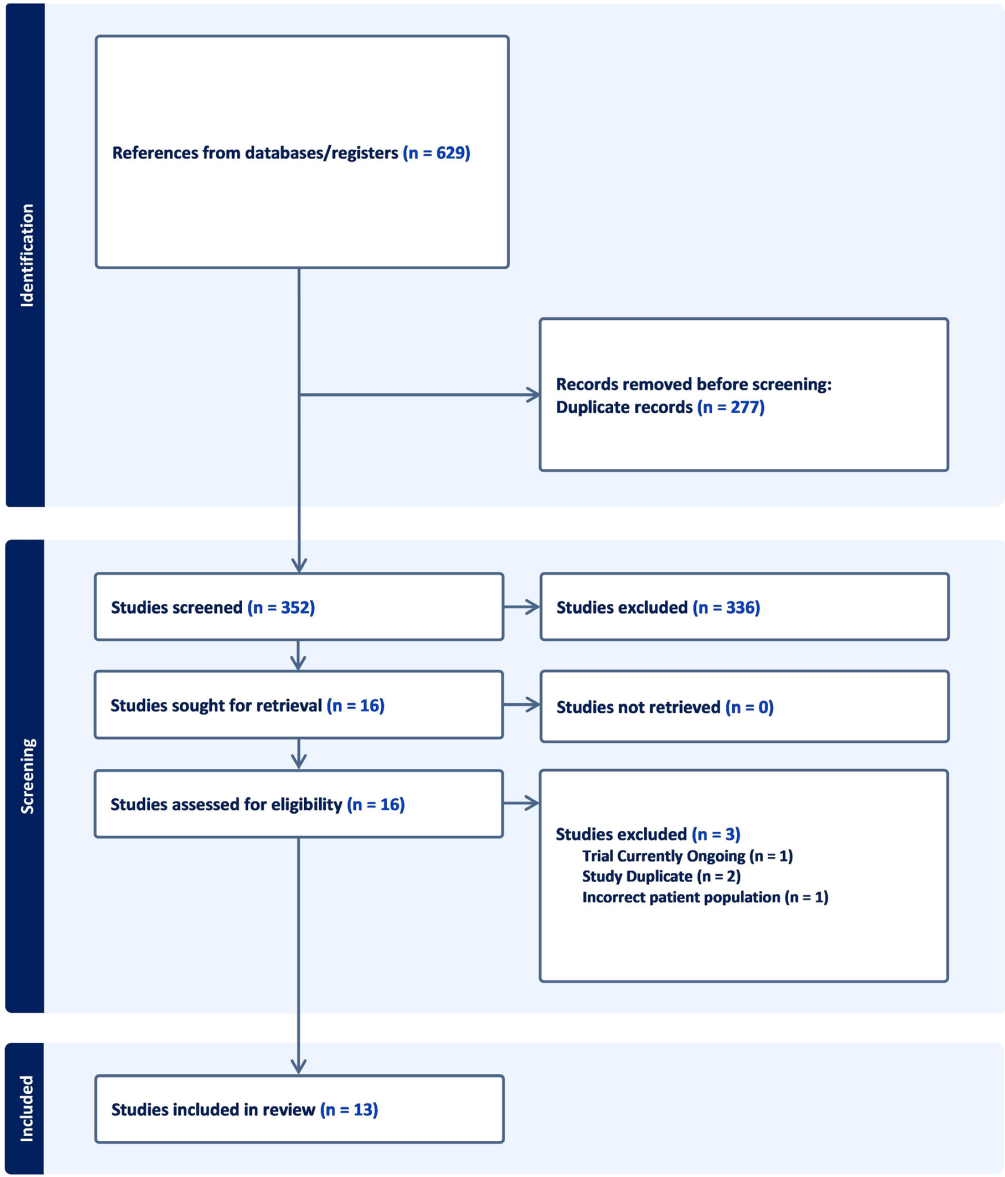
PRISMA flow diagram of study selection.

Selection of the studies entailed the independent screening of the titles and abstracts of all articles by the two independent assessors, A.S. and S.M.N. The full texts of all relevant articles were obtained and were screened once again by the independent reviewers. Any matters of discordance between the reviewers were addressed through discussion, with a third independent assessor, W.T., intervening if required. The process by which the articles were screened and selected was graphically depicted, seen in Figure 1, using the Preferred Reporting Items for Systematic Reviews and Meta‐Analyses (PRISMA) flow diagram.

### 2.11 Data Retrieval

The process encompassed documentation of the study methods, including study design, sample size and study duration. Additionally, characteristics of the study participants, details of interventions and comparators, measured outcomes, and sources of funding were also noted. The conflict of interest statements of the study authors were also recorded.

### 2.12 Risk of Bias Assessment of Included Studies

A.S. and S.M.N. evaluated the risk of bias of all studies included in an independent fashion whilst using the Cochrane risk‐of‐bias tool [24]. The risk of bias across seven domains (sequence generation, allocation concealment, blinding of participants and personnel, blinding of outcome assessment, incomplete outcome data, selective reporting and additional sources of bias) was classified as being low, of some concern, or high.

### 2.13 Measures of Treatment Efficacy

The study primarily examined long‐term outcomes relating to changes in mean CIMT. Additionally, the study also evaluated long‐term outcomes related to systemic vascular inflammation, glycaemic control, adverse events, metabolic parameters and cardiovascular risk factors, including HbA1c levels, lipid profiles, blood pressure and the incidence of cardiovascular events.

### 2.14 Management of Incomplete Data

All instances of incomplete or ambiguous information were systematically recorded, and efforts were made to contact the original authors to obtain any missing data.

### 2.15 Heterogeneity Assessment

As a result of the extensive criteria for inclusion, heterogeneity was rigorously assessed to ensure the validity and reliability of the reported outcomes.

### 2.16 Data Synthesis

Statistical analysis was conducted using the comprehensive web‐based AI analytical tool: Rayyan.ai Software [25].

### 2.17 Ethical Considerations

In conducting this review, formal ethical approval was not sought as such studies do not involve direct human participation. Nonetheless, the review adhered rigorously to established research methodologies, ensuring transparency in reporting and accuracy throughout the process.

## 3 Results

### 3.1 Description of Studies

A total of 13 RCTs was incorporated into the review.

### 3.2 Results of the Search

Following PRISMA guidelines a comprehensive search of five prominent databases of scientific literature identified a total of 629 articles (Figure 1). Prior to the screening of the articles, 277 duplicate records were identified and automatically excluded using the RayyanAI software, resulting in 352 remaining articles. Of these, 336 articles were independently reviewed and excluded by two reviewers in accordance with a set of predetermined criteria. Consequently, 16 studies were deemed potentially relevant and the full text of each was thoroughly screened. Upon further assessment of eligibility, the following three exclusions were made: one study which was classified as being in progress, one duplicate study and one study involving an unsuitable patient population. This screening process culminated in the final inclusion of 13 studies.

### 3.3 Included Studies

Thirteen RCTs are incorporated within this review:

1. The Campanian Postprandial Hyperglycaemia Study [26].
2. Effect of Pioglitazone on Carotid Intima‐Media Thickness and Arterial Stiffness in Type 2 Diabetic Nephropathy Patients (EPVS‐T2DN) [27].
3. Carotid Intima‐Media Thickness in Atherosclerosis Using Pioglitazone Trial [28] (CHICAGO Trial).
4. Peroxisome proliferator‐activated receptor gamma agonists for the Prevention of Adverse events following percutaneous coronary Revascularisation [29] (PPAR Study).
5. Pioglitazone anti‐atherosclerosis effect on prospective randomised open blinded end‐point trial [30] (PROBE Study).
6. Carotid Atherosclerosis: Metformin for insulin Resistance study [31] (CAMERA study).
7. Copenhagen Insulin and Metformin Therapy trial [32] (CIMT Trial).
8. Study of Preventive Effects of Alogliptin on Diabetic Atherosclerosis Trial [33] (SPEAD‐A Trial).
9. Sitagliptin Preventive Study of Intima‐Media Thickness Evaluation Trial [34] (SPIKE Trial).
10. Program of Vascular Evaluation under Glucose Control by DPP‐4 Inhibitor Study [35] (PROLOGUE Study).
11. Reducing with Metformin Vascular Adverse Lesions [36] (REMOVAL Trial).
12. Using Tofogliflozin for Possible better Intervention against Atherosclerosis for type 2 diabetes patients [37] (UTOPIA Trial).
13. Prevention of Atherosclerosis by SGLT2 inhibitor: a multicentre and randomised controlled study [38] (PROTECT Study).

Table 1 summarises the included studies characteristics including country, population (T2DM or CVD), sample size, age, sex distribution, drug versus comparator, dose, baseline CIMT (where available) and reference.

**TABLE 1:** Characteristics of trials included.

First author (year)/study abbreviation	Country	Duration (year)	Study pop.	Sample size (case/control)	Age (year)	Male %	Drug vs. comparator	Dose (mg/day)	Baseline mean CIMT—intervention (mm)	References
Esposito et al. (2004)/Campanian	Italy	1	T2DM	88/87 175	51.65 ± 6.15	93 (53.1%)	Repaglinide vs. Glyburide	1.5–12 vs. 5–20	0.849 ± 0.208	[26]
Nakamura et al. (2004)/EPVS‐T2DN	Japan	1	T2DM	15/15/15 45	64.6 ± 9.5	25 (55.6%)	Pioglitazone vs. Glibenclamide vs. Voglibose	30 vs. 5 vs. 0.6	0.76 ± 0.12	[27]
Mazzone et al. (2006)/CHICAGO Trial	America	1.4	T2DM	175/186 361	66.3 ± 8	230 (63.7%)	Pioglitazone vs. Glimepiride	15–45 vs. 1–4	0.771 ± 0.008	[28]
Bhatt et al. (2007)/PPAR Study	USA and Australia	1	CVD	102/98 200	59.4 ± 9.7	160 (80%)	Rosiglitazone vs. Placebo	8 vs. 0	Unreported	[29]
Yamasaki et al. (2010)/PROBE Study	Japan	4	T2DM	89/97 186	56.9	117 (62.9%)	Pioglitazone vs. Placebo	15–45 vs. 0	0.839 ± 0.1873	[30]
Preiss et al. (2014)/CAMERA study	UK	1.5	CVD	86/87 173	63.5 ± 8	133 (76.9%)	Metformin vs. Placebo	1700 vs. 0	0.712 ± 0.126	[31]
Lundby‐Christensen et al. (2016)/CIMT Trial	Denmark	1.5	T2DM	206/206 412	60.65 ± 8.9	281 (68.2%)	Metformin vs. Placebo	2000 vs. 0	0.788 ± 0.135	[32]
Mita et al. (2016)/SPEAD‐A Trial	Japan	2	T2DM	172/169 341	64.6 ± 9.5	199 (58.4%)	Alogliptin vs. Placebo	25 vs. 0	0.83 ± 0.15	[33]
Mita et al. (2016)/SPIKE Trial	Japan	2	T2DM	142/140 282	63.7 ± 5	165 (58.5%)	Sitagliptin vs. Placebo	25–100 vs. 0	0.84 ± 0.19	[34]
Oyama et al. (2016)/PROLOGUE Study	Japan	2	T2DM	222/220 442	63.9 ± 9	297 (67.2%)	Sitagliptin vs. Placebo	25–100 vs. 0	0.829 ± 0.166	[35]
Petrie et al. (2017)/REMOVAL Trial	Multi‐country	3	T1DM	219/209 428	55.5 ± 8.5	253 (59.1%)	Metformin vs. Placebo	2000 vs. 0	0.773 ± 0.140	[36]
Katakami et al. (2020)/UTOPIA Trial	Japan	2	T2DM	169/171 340	61.1 ± 9.5	198 (58.4%)	Tofogliflozin vs. Placebo	20 vs. 0	0.87 ± 0.16	[37]
Tanaka et al. (2023)/PROTECT Study	Japan	2	T2DM & CVD	232/232 464	68 ± 5.9	161 (69.4%)	Ipragliflozin vs. Placebo	50–100 vs. 0	0.79 (0.705, 0.905)	[38]

Abbreviations: CIMT, carotid intima‐media thickness; CVD, cardiovascular disease; T2DM, type 2 diabetes mellitus.

### 3.4 Medication Family Grouping

The studies included in this review examined several families of medications:

#### 3.4.1 Thiazolidinediones (TZDs)

- Pioglitazone was assessed in the EPVS‐T2DN [27], CHICAGO Trial [28], PROBE Study [30] and indirectly in combination with other drugs in the PPAR Study [29].
- Rosiglitazone was specifically studied in the PPAR Study [29].

#### 3.4.2 Biguanides

- Metformin was investigated in the CAMERA Study [31], CIMT Trial [32] and the REMOVAL Trial [36].

#### 3.4.3 Dipeptidyl Peptidase‐4 (DPP‐4) Inhibitors

- Sitagliptin was the focus of the SPIKE Trial [34] and PROLOGUE Study [35].
- Alogliptin was studied in the SPEAD‐A Trial [33].

#### 3.4.4 Sodium‐Glucose Cotransporter‐2 (SGLT2) Inhibitors

- Tofogliflozin was explored in the UTOPIA Trial [37].
- Ipragliflozin was examined in the PROTECT Study [38].

#### 3.4.5 Other Hypoglycaemic Agents

- Repaglinide, a meglitinide analogue, was compared to glyburide in the Campanian Study [26].

### 3.5 Participants

The Campanian Postprandial Hyperglycaemia Study, initially an epidemiological study, transitioned into an RCT involving drug‐naïve type 2 diabetes patients from two Southern Italian towns. Participants were aged 35–70, had a diabetes duration of 3 years or less, a BMI of 24 or higher, an HbA1c of 6.5% or greater and were managed through diet or OHAs.

The CAMERA trial included participants aged 35–75 years with diagnosed coronary heart disease, a large waist circumference as per International Diabetes Foundation criteria, and statin use. The REMOVAL trial, conducted in five Western countries, focused on type 1 diabetics aged 40 or older with at least a 5‐year history and three or more ASCVD risk factors. The CIMT trial, unlike other metformin trials, did not consider cardiovascular risk factors and included type 2 diabetics treated with OHAs for at least a year or insulin for 3 months, excluding those with over 70% carotid artery stenosis.

The PROTECT study included adults with HbA1c levels of 6%–10%, resistant to diet or exercise, who had started conventional diabetic therapy at least 3 months before the trial. Exclusion criteria included any history of ASCVD and recent initiation of an SGLT‐2 inhibitor. The UTOPIA trial focused on Japanese individuals with uncontrolled T2DM (HbA1c ≥ 6% and < 9%), resistant to diet, exercise or medication, excluding recent SGLT‐2 inhibitor users. Standard exclusion criteria applied, with some discretion allowed to investigators. Detailed inclusion criteria for both studies are available in their supplementary materials [39].

The PROLOGUE trial included T2DM patients aged 30 or older with HbA1c levels between 6.2% and 9.4%, unresponsive to diet, exercise or standard anti‐diabetic agents. Exclusions included those using other DPP‐4is, GLP‐1 analogues and individuals with advanced heart failure, based on New York Heart Association functional classes. The SPIKE and SPEAD‐A trials used similar criteria, defining uncontrolled blood glucose according to the 2010 Japanese Diabetes Society guidelines and excluding those on medications interacting with incretin‐based treatments. Detailed inclusion and exclusion criteria are provided in the Research Design and Methods sections of the respective studies.

Nakamura et al. [27] provided limited details on inclusion and exclusion criteria but mentioned using similar HbA1c cut‐offs and unique fasting C‐peptide level criteria. The PPAR study included participants aged 21–85 undergoing elective or urgent PCI with coronary artery disease, requiring obesity and at least one risk factor like hypertension, dyslipidaemia or elevated HbA1c/glucose levels. Exclusions were recent PCI, significant heart failure or diabetes needing recent treatment. The CHICAGO trial focused on adults with T2DM, requiring HbA1c levels of 6.5%–9% (or up to 10% if untreated), excluding severe cardiovascular conditions, liver disease and significant obesity. It included a multi‐ethnic cohort. The PROBE trial involved adults aged 35–74 with T2DM (HbA1c ≥ 6.5%) and at least two risk factors such as hypertension, dyslipidaemia, obesity or smoking. Exclusions were type 1 diabetes, severe cardiovascular or renal/hepatic impairment and recent major cardiovascular events. These studies primarily targeted individuals with high cardiovascular risk or specific metabolic conditions, ensuring a focused and relevant population for assessing the effects of OHAs on CIMT.

### 3.6 Intervention

Collectively, the 13 RCTs involved a pool of 3849 participants. The nature of the various populations, interventions and control groups contained within each study is detailed in Table 1.

### 3.7 Outcomes

The reviewed trials primarily focused on changes in CIMT as a key outcome, with secondary outcomes including markers of glycaemic control, lipid profiles, renal function and cardiovascular risk factors. Trials such as CIMT, REMOVAL, CAMERA, PROTECT and UTOPIA measured mean CIMT changes and additional cardiovascular health markers. The SPEAD‐A, SPIKE and other trials also examined glycaemic control, nephropathy and adverse cardiovascular events. Overall, these studies provide valuable insights into the effectiveness and safety of various OHAs in managing diabetes and ASCVD.

### 3.8 Excluded Studies

The SAMAS trial, a single‐blinded, randomised study, is investigating the effects of 12 months of oral semaglutide therapy (14 mg) on ASCVD risk factors in patients with type 2 diabetes. With 3183 participants, the trial's primary outcomes include reductions in CIMT, improved endothelial function and decreased arterial stiffness. Secondary outcomes include changes in lipid levels, HbA1c and high‐sensitivity C‐reactive protein. However, since the trial is still in its third phase and results have not yet been released, it was excluded from the review [40].

### 3.9 Assessment of Bias Risk in Included Studies

**FIGURE 2:**
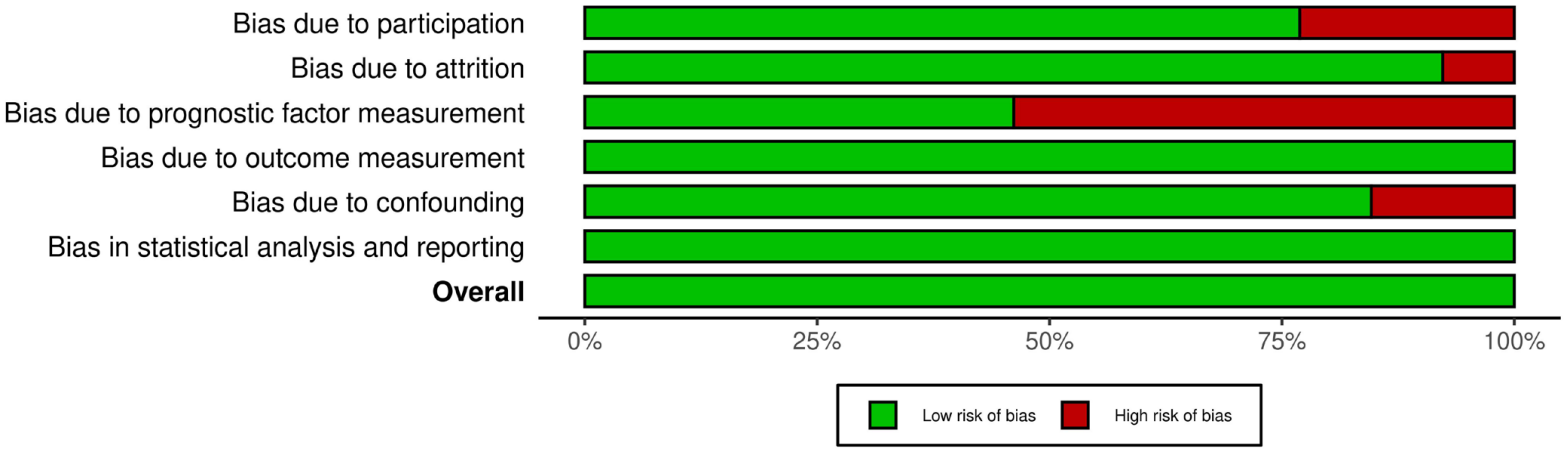
Summary plot of risk of bias.

**FIGURE 3:**
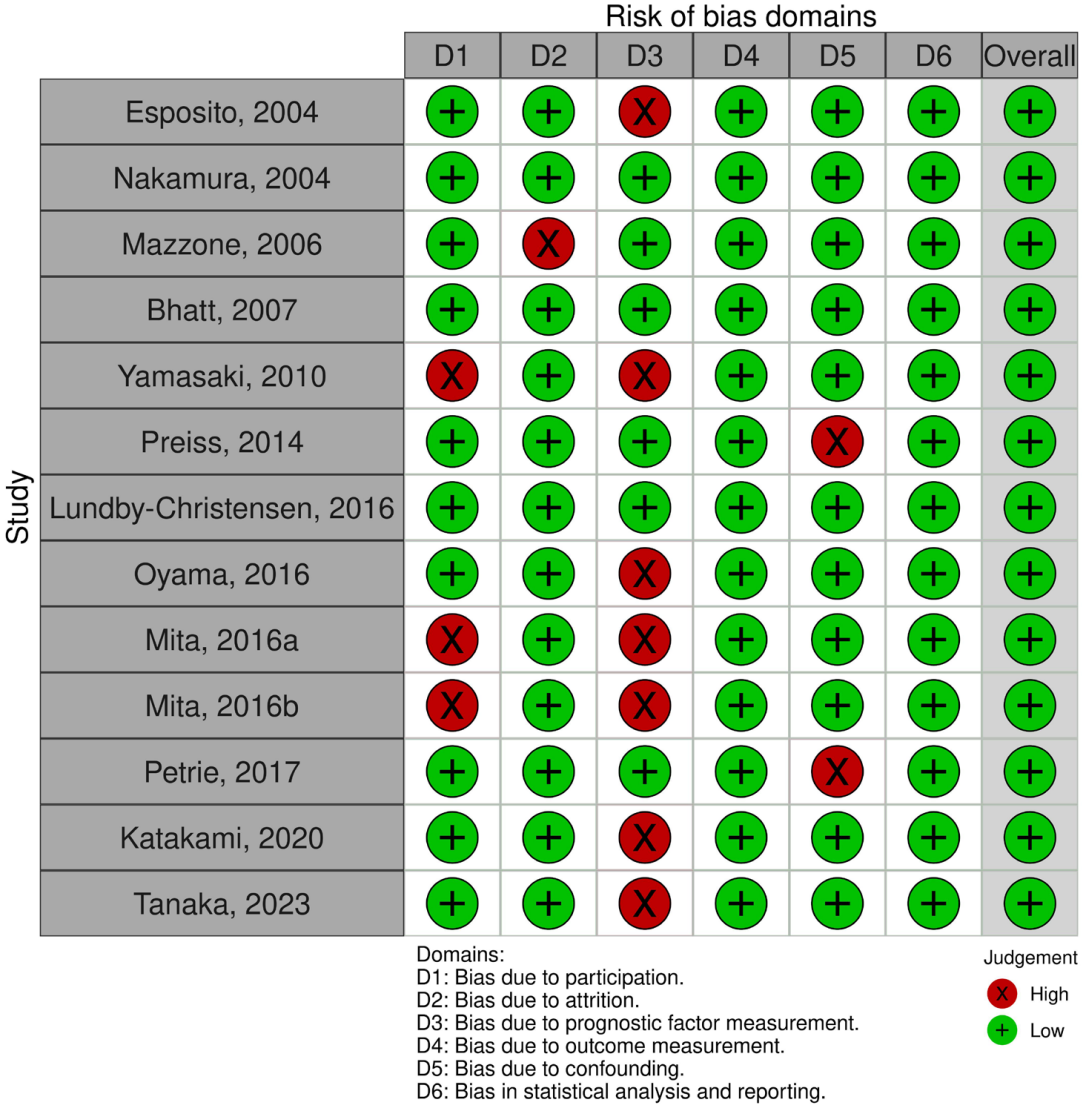
Risk of bias traffic light plot.

The assessment of bias risk is illustrated in Figures 2 and 3.

Figure 2 presents the proportion of studies assessed as having a low or high risk of bias across six domains: bias due to participation, attrition, prognostic factor measurement, outcome measurement, confounding and statistical analysis/reporting. Green bars represent the proportion of studies with a low risk of bias, while red bars indicate a high risk of bias. The overall risk of bias is also summarised at the bottom. Five out of six domains demonstrated low risk with prognostic factor measurement showing higher levels of potential bias.

Figure 3 shows the risk of bias assessment across six domains. Each domain (D1–D6) represents a specific type of bias, judged as low (green) or high (red) risk. The overall risk of bias is shown in the final column.

### 3.10 Allocation/Selection Bias

The PROBE, SPIKE and SPEAD‐A trials employed a dynamic allocation method based on prespecified characteristics and consequently randomisation of sequence generation could not be ensured.

However, all other studies offered satisfactory descriptions of the process of randomisation and allocation of participants and were therefore deemed to be of low risk for selection bias.

### 3.11 Blinding; Detection and Performance Biases

All 13 trials reported blinding of the assessment of the primary and secondary outcomes. The Campanian Postprandial Hypoglycaemic Study, CIMT, PROTECT, UTOPIA, PROLOGUE, SPIKE, SPEAD‐A and PROBE were performed using an open‐label approach and therefore the patients involved were not blinded.

### 3.12 Incomplete Outcome Data/Attrition Bias

All papers, besides the CHICAGO trial, were reported as being of minimal risk of attrition bias due to negligible dropouts reported.

The CHICAGO trial demonstrated a dropout rate of 30%; however, the dropout rate was balanced between the two treatment groups and a subanalysis of those who dropped out showed no significant variation from those who remained. This indicates that the risk of attrition bias may not skew the results significantly.

### 3.13 Selective Reporting/Reporting Bias

All included papers reported predetermined study outcomes, suggesting a minimal risk of bias in the reporting of results.

### 3.14 Other Sources of Bias

We could not identify any alternative sources of potential bias within the included papers.

Table 2 summarises the funding sources for each of the included studies. Most studies reported receiving financial or institutional support, with funding commonly provided by pharmaceutical companies such as Takeda Pharmaceuticals, Astellas Pharma, Novo Nordisk and GlaxoSmithKline. Some trials, including CAMERA and CIMT, were supported through independent or public research grants, while one study (EPVS‐T2DN) did not specify its funding source. This information provides transparency regarding potential financial involvement and complements the overall assessment of study quality.

**TABLE 2:** Sources of funding for included randomised controlled trials.

First author (year)/study abbreviation	Funded?	Funding organisation/company (grant name)	References
Esposito et al. (2004)/Campanian	Yes	Second University of Naples, Regione Campania	[26]
Nakamura et al. (2004)/EPVS‐T2DN	Not stated explicitly	—	[27]
Mazzone et al. (2006)/CHICAGO Trial	Yes	Takeda Pharmaceuticals, National Heart, Lung, Blood institute NHLBI (K25 HL68139‐01A1)	[28]
Bhatt et al. (2007)/PPAR Study	Yes	GlaxoSmithKline (GSK)	[29]
Yamasaki et al. (2010)/PROBE Study	Yes	Takeda Pharmaceuticals	[30]
Preiss et al. (2014)/CAMERA study	Yes	Chief Scientific Office Scotland	[31]
Lundby‐Christensen et al. (2016)/CIMT Trial	Yes	Unrestricted grant from Novo Nordisk	[32]
Mita et al. (2016)/SPEAD‐A Trial	Yes	Astellas Pharma, AstraZeneca, Bayer, etc.	[33]
Mita et al. (2016)/SPIKE Trial	Yes	Mitsubishi Tanabe Pharma, Ono, Novo Nordisk	[34]
Oyama et al. (2016)/PROLOGUE Study	Yes	Research grant from Clinical Research Promotion Foundation (No. 1026)	[35]
Petrie et al. (2017)/REMOVAL Trial	Yes	Primary: Strategic Research Award 17‐2011‐272 from Juvenile Diabetes Research Foundation In‐kind support: Merck, KGaA and Itamar Medical	[36]
Katakami et al. (2020)/UTOPIA Trial	Yes	Kowa Company	[37]
Tanaka et al. (2023)/PROTECT Study	Yes	Astellas Pharma	[38]

### 3.15 Primary Outcome

Table 3 presents the primary outcomes reported across the included studies. The table details the intervention and comparator drugs, baseline mean CIMT values, the reported mean change in CIMT with corresponding standard deviations and confidence intervals where available, the direction of change and statistical significance for each study.

**TABLE 3:** Primary outcomes of trials included.

First author (year)/study abbreviation	Drug	Comparator	Baseline mean CIMT—intervention (mm)	ΔCIMT (mean ± SD)	95% CI	Direction of change	*p*/significance	References
Esposito et al. (2004)/Campanian	Repaglinide	Glyburide	0.849 ± 0.208	0.029 ± 0.021 in Repaglinide group/0.005 ± 0.01 in Glyburide group	−0.041 to −0.017 in Repaglinide group/−0.010 to 0.005 in Glyburide group	Decreased	0.02	[26]
Nakamura et al. (2004)/EPVS‐T2DN	Pioglitazone	Glibenclamide/Voglibose	0.76 ± 0.12	0.08 mm in Pioglitazone group but no significant change in Glibenclamide and Voglibose groups	Not mentioned	Decreased	< 0.05	[27]
Mazzone et al. (2006)/CHICAGO Trial	Pioglitazone	Glimepiride	0.771 ± 0.008	0.013	−0.024 to −0.002	Decreased	0.02	[28]
Bhatt et al. (2007)/PPAR Study	Rosiglitazone	Placebo	Unreported	0.013 ± 0.02	−0.02 to 0.02	Decreased	0.49	[29]
Yamasaki et al. (2010)/PROBE Study	Pioglitazone	Placebo	0.839 ± 0.1873	0.058 ± 0.1718 in Pioglitazone Group 0.043 ± 0.1644 in control group	—	Decreased	Not significant	[30]
Preiss et al. (2014)/CAMERA study	Metformin	Placebo	0.712 ± 0.126	0.007	−0.006 to 0.020	Increased	0.29	[31]
Lundby‐Christensen et al. (2016)/CIMT Trial	Metformin	Placebo	0.788 ± 0.135	0.012	−0.003 to 0.026	No change	0.11	[32]
Mita et al. (2016)/SPEAD‐A Trial	Alogliptin	Placebo	0.83 ± 0.15	0.030	−0.057 to −0.004	Decreased	0.022	[33]
Mita et al. (2016)/SPIKE Trial	Sitagliptin	Placebo	0.829 ± 0.166	0.053	−0.090 to −0.016	Decreased	0.005	[34]
Oyama et al. (2016)/PROLOGUE Study	Sitagliptin	Placebo	0.84 ± 0.19	0.009	−0.028 to 0.011	Decreased	0.309	[35]
Petrie et al. (2017)/REMOVAL Trial	Metformin	Placebo	0.773 ± 0.140	0.0167	−0.012 to 0.002	Increased	0.166	[36]
Katakami et al. (2020)/UTOPIA Trial	Tofogliflozin	Placebo	0.87 ± 0.16	0.008	−0.009 to 0.025	Decreased	0.34	[37]
Tanaka et al. (2023)/PROTECT Study	Ipragliflozin	Placebo	0.8195 ± 0.7950–0.8441	0.0001	−0.0191 to 0.0189	No significant change	0.989	[38]

In studies where mean change data were not explicitly provided, the available intervention and comparator results have been listed. The table illustrates that thiazolidinediones and DPP‐4 inhibitors generally showed reductions in CIMT, whereas metformin and SGLT‐2 inhibitors demonstrated neutral or minimal effects.

The Campanian Postprandial Hyperglycaemia Study found that Repaglinide led to greater CIMT regression compared to Glyburide, with approximately half of the Repaglinide group experiencing regression versus only 18% in the Glyburide group. This suggests that Repaglinide offers better cardiovascular protection.

The CAMERA trial, which compared Metformin to a placebo over 18 months in individuals with established ASCVD, found no effect of Metformin on CIMT in non‐diabetics at high cardiovascular risk, as both groups showed significant CIMT progression. Similarly, the REMOVAL trial, which studied Metformin in type 1 diabetes patients, also reported no significant difference in CIMT between Metformin and placebo. The CIMT trial in type 2 diabetes patients on insulin mirrored these findings, with no significant change in CIMT between the Metformin and placebo groups.

The PROTECT trial, examining Ipragliflozin, found no significant change in CIMT compared to the control group, although subgroup analysis hinted at a potential benefit in patients on statins. This was consistent with findings from the UTOPIA trial, which reported no significant difference in CIMT change between Tofogliflozin and conventional therapy.

In the PROLOGUE trial, Sitagliptin showed no significant difference in CIMT compared to conventional therapy in type 2 diabetes patients. However, the SPIKE trial suggested that Sitagliptin might slow the progression of certain CIMT measurements. The SPEAD‐A study reported that Alogliptin significantly reduced and prevented CIMT progression.

Pioglitazone, compared to Glibenclamide and Voglibose, was found to significantly reduce CIMT in a Japanese study. The CHICAGO trial also favoured Pioglitazone, reporting a slight reduction in CIMT compared to an increase in the Glimepiride group. The PPAR study found no significant CIMT difference with Rosiglitazone, while the PROBE study observed CIMT regression with Pioglitazone, although the difference was not statistically significant.

As a formal meta‐analysis was not feasible due to the heterogeneity of study designs, interventions and outcome measures, a descriptive synthesis was undertaken to summarise the direction and magnitude of CIMT change across drug classes. The pertinent data has been described where available; however for RCTs where these values are not available, the intervention and comparator statistics have been detailed. Thiazolidinediones, particularly pioglitazone, demonstrated consistent reductions in CIMT in EPVS‐T2DN, CHICAGO and PROBE, with mean decreases of approximately 0.01–0.04 mm compared with baseline. DPP‐4 inhibitors showed modest improvement, with alogliptin in SPEAD‐A and sitagliptin in SPIKE each reducing CIMT by roughly 0.03–0.05 mm, while PROLOGUE found no significant difference. In contrast, metformin yielded neutral results in CAMERA, CIMT and REMOVAL, and SGLT‐2 inhibitors such as tofogliflozin and ipragliflozin in UTOPIA and PROTECT showed no measurable change. Repaglinide, examined in the Campanian Study, was associated with a small but statistically significant reduction compared with Glibenclamide. These findings are summarised narratively in a descriptive table to facilitate comparison between drug classes and illustrate the overall direction of vascular change.

In summary, certain OHAs, particularly Repaglinide and Pioglitazone, demonstrate a more favourable impact on CIMT, suggesting potential cardiovascular benefits in patients with ASCVD and/or diabetes.

### 3.16 Secondary Outcomes

The secondary outcomes of the systematic review reveal varying impacts of OHAs on glycaemic control, cardiovascular markers and adverse events in patients with diabetes and/or cardiovascular disease.

In the Campanian Postprandial Hyperglycaemia Study, both Repaglinide and Glibenclamide effectively reduced HbA1c, but Repaglinide showed superior outcomes in lowering postprandial glucose peaks and inflammatory markers, indicating greater cardiovascular protection in T2DM patients.

Metformin, studied in the CAMERA, REMOVAL and CIMT trials, consistently reduced measures of adiposity and HbA1c. However, its impact on other cardiovascular markers like cholesterol and fasting glucose was limited. Metformin was also associated with a higher incidence of gastrointestinal side effects, though serious adverse events were infrequent.

The PROTECT and UTOPIA trials on SGLT‐2is (Ipragliflozin and Tofogliflozin) found significant improvements in HbA1c, blood pressure and other metabolic parameters. However, neither trial showed significant differences in MACE between the treatment and conventional therapy groups.

DPP‐4is, such as Sitagliptin and Alogliptin, demonstrated enhanced glycaemic control in the PROLOGUE, SPIKE and SPEAD‐A trials, with Sitagliptin and Alogliptin reducing HbA1c levels significantly. However, cardiovascular risk factors and adverse events were similar between treatment and conventional therapy groups.

Pioglitazone, highlighted in trials like CHICAGO and Nakamura et al., was particularly effective in reducing urinary albumin excretion, enhancing HDL cholesterol levels and decreasing triglycerides, demonstrating benefits beyond glycaemic control. However, it was associated with weight gain and an increased risk of oedema. In contrast, Rosiglitazone, studied in the PPAR and PROBE trials, showed minimal impact on cardiovascular events but did improve lipid profiles and reduce the likelihood of developing diabetes.

Overall, the results indicate that while OHAs like Repaglinide and Pioglitazone offer notable cardiovascular and metabolic benefits, others, such as Metformin and DPP‐4is, are more limited in their effects on cardiovascular markers, highlighting the need for tailored therapeutic approaches.

## 4 Discussion

This review evaluates the efficacy of various OHAs in reducing CIMT and, by extension, attenuating ASCVD in patients with DM and/or established cardiovascular disease. The review highlights the significant role OHAs can play in preventing the progression of ASCVD, although their effectiveness varies.

Metformin, a first‐line treatment for T2DM, is well documented for improving cardiovascular outcomes and reducing HbA1c [41]. However, trials like CIMT, REMOVAL and CAMERA indicate that metformin has minimal impact on CIMT progression. These findings suggest that while metformin may confer cardiovascular benefits, these are likely due to its metabolic effects rather than direct changes in vascular structure.

The Campanian Postprandial Hyperglycaemia Study highlights the superiority of repaglinide over glibenclamide in reducing CIMT, emphasising the importance of targeting postprandial hyperglycaemia in reducing ASCVD risk in diabetics. This suggests that different OHAs have distinct impacts on cardiovascular outcomes, necessitating tailored treatment approaches.

SGLT‐2is, such as Tofogliflozin and Ipragliflozin, have gained attention for their cardioprotective properties, particularly in diabetic cardiomyopathy and heart failure [42]. However, trials like UTOPIA and PROTECT showed no significant reduction in CIMT progression with these drugs, indicating that their cardiovascular benefits may arise from improving endothelial function rather than altering vascular architecture.

DPP‐4is, including Sitagliptin and Alogliptin, have shown promise in reducing CIMT in some trials, such as SPEAD‐A and SPIKE. These findings suggest that DPP‐4is may reduce atherosclerosis by suppressing inflammatory processes. However, the PROLOGUE study's contrasting results, where Sitagliptin did not reduce CIMT compared to other OHAs, highlight the complexity of interpreting these effects.

Thiazolidinediones, particularly pioglitazone, have demonstrated mixed results. While the CHICAGO trial and studies on diabetic nephropathy patients showed significant CIMT reduction with pioglitazone, the PPAR study found no such effect with rosiglitazone. These discrepancies may be due to differences in study populations, design and the specific drugs used. Despite the associated risk of heart failure, pioglitazone's consistent reduction in CIMT suggests it may be a favourable option for reducing ASCVD burden in diabetics, as acknowledged in the latest ESC guidelines [43, 44].

The variability among the included studies must be considered when interpreting these results. The trials encompassed participants with both type 1 and type 2 diabetes, as well as individuals with and without established ASCVD. These differences influence both baseline risk and response to treatment. For instance, the REMOVAL trial, which involved adults with T1DM, demonstrated no meaningful effect of metformin on CIMT, while studies such as CHICAGO, EPVS‐T2DN and PROBE, conducted in T2DM populations, found that pioglitazone slowed CIMT progression compared with control agents. Trials enrolling patients with established atherosclerotic disease, including CAMERA and PPAR, generally reported smaller or neutral effects, suggesting that CIMT modification may be more evident in earlier disease stages. Differences in follow‐up duration, which ranged from 6 months to 4 years, may also account for the variability in the magnitude of effect. Collectively, these observations indicate that the results of this review are most applicable to patients with T2DM and co‐existing cardiovascular disease, who comprised the majority of participants in the included randomised controlled trials.

In conclusion, the review underscores the nuanced benefits of OHAs in reducing CIMT and ASCVD risk. While drugs like metformin and SGLT‐2is offer cardioprotective benefits, their impact on CIMT is limited, indicating that their effects may be mediated through mechanisms other than vascular remodelling. On the other hand, pioglitazone's significant impact on CIMT suggests it could be a valuable part of a comprehensive strategy to reduce cardiovascular events, despite its heart failure risk. These findings advocate for a tailored approach to diabetes management, considering both glycaemic control and the broader cardiovascular profile of the chosen OHA. Further research is needed to refine these strategies and optimise patient outcomes.

### 4.1 Summary of Main Results

This analysis emphasises the well‐recognised role of anti‐diabetic medication in the risk reduction and management of ASCVD. While illustrating the profound impact on the progression of CIMT and thereby the risk of ASCVD and ASCVD‐related events, the included RCTs elucidate the distinct mechanisms and clinical effects of various classes of OHAs. Consequently, it can be appreciated that certain OHAs effectuate appreciable changes in the progression of CIMT and the management of ASCVD. These results highlight the necessity for a more integrated management strategy, regular monitoring of CIMT, and further research to enhance therapeutic strategies for DM and/or ASCVD patients.

### 4.2 Limitations

This review has several limitations. The small number of studies included some with limited sample sizes, may affect the accuracy and generalisability of the findings. The review covers various classes of hypoglycaemic agents, each with distinct mechanisms of action, which may produce different outcomes in diverse populations with ASCVD and/or DM. The review is also limited by its focus on patients with type 1 DM, type 2 DM and cardiovascular disease, excluding those with other metabolic syndromes, thereby narrowing its scope.

Variations in CIMT measurements due to differences in ultrasound machines and methodologies, such as whether plaque‐free segments or plaque lesions were included, further complicate comparisons. Additionally, the studies varied significantly in participant demographics, the use of concurrent hypoglycaemic agents, follow‐up durations and secondary outcomes assessed, resulting in notable heterogeneity. These factors should be carefully considered when interpreting the results and deriving conclusions from this review.

Several of the included trials, particularly those conducted in Japan such as SPEAD‐A, SPIKE, PROLOGUE, UTOPIA and PROTECT, used open‐label designs, which may increase the risk of performance bias. As CIMT was measured by trained sonographers who were blinded to treatment allocation, the influence of observer bias is likely to have been minimal. Nevertheless, open‐label protocols may affect treatment adherence, concurrent medication use and the reporting of adverse effects. In addition, limited reporting of allocation concealment in studies such as PPAR and PROBE may introduce a degree of selection bias. Overall, most studies demonstrated a low to moderate risk of bias across key domains, and the objectivity of CIMT measurement supports the credibility of the overall findings.

### 4.3 Congruence and Disagreement With Existing Literature

Several systematic reviews and meta‐analyses have been performed to evaluate the use of hypoglycaemic agents in influencing CIMT progression in DM and ASCVD patients. The majority of these have primarily focused on the use of Metformin among this specific demographic [45]. Our findings of no statistical significance between Metformin and conventional therapy in reducing CIMT progression are not in alignment with past studies evaluating this medication [46, 47]. However, our findings pertaining to the efficacy of Thiazolidinediones and DPP‐4is in attenuating CIMT progression are congruent with the existing body of literature in many ways. The immense benefits of utilising thiazolidinediones and DPP‐4is in those with or at risk of ASCVD observed in previous studies resonate with our findings, underscoring the multifaceted benefits of OHAs [48, 49]. This congruence reinforces the idea that the use of select OHAs in DM and ASCVD patients can be a viable and multifaceted strategy in reducing overall cardiovascular risk.

## 5 Conclusions

Our systematic review concluded that the use of OHAs in patients with diabetes and/or ASCVD has a variable impact on CIMT, highlighting the complex nature of OHAs in ASCVD. While some OHAs, such as Pioglitazone, were associated with a reduction in CIMT, others such as metformin showed negligible effects. The reviewed RCTs collectively emphasise the necessity for a more integrated approach in the management of ASCVD, acknowledging that while all OHAs offer significant potential in managing glucose levels in DM and ASCVD patients, the choice of OHA may influence cardiovascular outcomes in those with or at risk of ASCVD, particularly through reducing CIMT progression and consequently attenuating a patient's overall cardiovascular risk profile. In conclusion, the prolonged use of OHAs in those with DM and ASCVD holds promise in the enhancement of cardiovascular outcomes as part of a comprehensive ASCVD risk reduction strategy.

### 5.1 Implications for Clinical Practice

Our systematic review highlights the critical role of hypoglycaemic therapies in modulating hyperglycaemia, a known RF that predisposes to ASCVD, and ASCVD itself. Firstly, our findings of certain OHAs yielding superior results in the attenuation of CIMT may influence clinicians to select certain therapies that not only control glucose levels but also address cardiovascular risk among those with DM and known ASCVD. Additionally, these results emphasise the need for a more integrated approach to managing diabetes and ASCVD, thereby improving patient outcomes and quality of life by prioritising medications with dual benefits.

### 5.2 Implications for Future Research

This review highlights the need for further research to better understand the effects of OHAs on CIMT in patients with ASCVD and/or and DM. Future studies should focus on elucidating the mechanisms through which specific OHAs influence CIMT, and the pathways involved in atherosclerosis. Long‐term comparative studies are necessary to assess the sustained benefits and potential adverse effects of different OHAs, including newer therapies like SGLT‐2is, DPP‐4is and GLP‐1 receptor agonists. Research into the cardiovascular outcomes of emerging OHAs, such as semaglutide, is also crucial [50]. Additionally, the impact of combined OHA therapies on CIMT is currently under‐researched and warrants further investigation to optimise treatment strategies for high‐risk patients. Addressing these gaps could significantly enhance clinical outcomes for those with DM and ASCVD.

## Author Contributions

**Ali Shabu:** conceptualisation, investigation, methodology, software, article screening, data curation, formal analysis, writing – original draft, writing – review and editing. **Syed Mohammad Naqvi:** investigation, methodology, software, article screening, data curation, formal analysis, supervision, writing – review and editing. **Farshad Hesari:** writing – review and editing. **Syed Yaseen Naqvi:** writing – review and editing. **Wael Tawfick:** conceptualisation, methodology, supervision.

## Funding

The authors have nothing to report.

## Conflicts of Interest

The authors declare no conflicts of interest.

## Data Availability

The data that support the findings of this study are available from the corresponding author upon reasonable request.
